# Treadmill and Running Speed Effects on Acceleration Impacts: Curved Non-Motorized Treadmill vs. Conventional Motorized Treadmill

**DOI:** 10.3390/ijerph18105475

**Published:** 2021-05-20

**Authors:** Alberto Encarnación-Martínez, Ignacio Catalá-Vilaplana, Rafael Berenguer-Vidal, Roberto Sanchis-Sanchis, Borja Ochoa-Puig, Pedro Pérez-Soriano

**Affiliations:** 1 Research Group in Sports Biomechanics (GIBD), Department of Physical Education and Sports, University of Valencia, 46010 Valencia, Spain; Ignacio.catala@uv.es (I.C.-V.); roberto.sanchis@ua.es (R.S.-S.); boropuig@alumni.uv.es (B.O.-P.); pedro.perez-soriano@uv.es (P.P.-S.); 2 Grupo de Investigación en Telecomunicaciones Avanzadas (GRITA), Catholic University of Murcia, 30107 Guadalupe, Spain; rberenguer@ucam.edu; 3 Physical Education and Sport, University of Alicante, 03690 San Vicente del Raspeig, Spain

**Keywords:** biomechanics, accelerometer, treadmill, locomotion

## Abstract

An increase in the popularity of running can be seen over the last decades, with a large number of injuries on it. Most of the running injuries are related to impact accelerations and are due to overuse. In order to reduce the risk of injury or to improve performance and health new treadmill designs have been created, as it can be the curved non-motorized treadmill. The aim of this study was to analyse impact accelerations, spatio-temporal parameters and perceptual differences while running on curved non-motorized treadmill (cNMT) compared to motorized treadmill (MT) at different speeds. Therefore, 27 recreational runners completed two tests consisting of 10 min warm-up and three bouts of 8 min running at 2.77 m/s, 3.33 m/s and self-selected speed on cNMT and MT, previously randomised. Although the surface did not influence spatio-temporal parameters, a reduction in impact accelerations, head acceleration rate (mean effect size [ES] = 0.86), tibia peak (mean ES = 0.45) and tibia magnitude (mean ES = 0.55), was observed while running on cNMT in comparison with running on MT. Moreover, higher heart rate (HR) (mean ES = 0.51) and rating of perceived effort (RPE) (mean ES = 0.34) were found while running on cNMT. These findings demonstrated that higher intensity training and lower impact accelerations are experimented on cNMT, what can be used by trainers and athletes while planning training sessions.

## 1. Introduction

The popularity of running has been increasing during the last years due to its benefits for health, accessibility and low cost, becoming one of the most common ways to exercise [1]. Despite its numerous benefits, an increase in the prevalence of running injuries can be observed. It has been suggested that around the 42.7% of runners will get injured each year [2], being the majority of this injuries due to overuse [3].

Repeated and accumulated exposure to impact accelerations during long-distance running can overload and fatigue the musculoskeletal system, reducing its ability to absorb them and increasing the risk of injury [4]. As a result, impact acceleration analysis has received considerable scientific interest in order to reduce these accelerations during running and decrease the incidence of overuse injuries [5].

Several factors can affect these impact accelerations during running, including stride parameters (length and frequency) [6], fatigue [7,8], running mechanics [9], foot strike pattern [10], sports equipment (footwear, compression socks or plantar supports) [11], running surface [12,13,14] and running speed [15,16,17,18].

Among all the factors that can influence impact accelerations, running surfaces have been shown to influence acceleration impacts during: overground running vs. motorized treadmill [7,12,13], concrete vs. grass [14], woodchip trail vs. synthetic track/concrete [15]. On the other hand, it has been shown that impact accelerations increase with higher velocities [17,18].

Runners, whenever they can and if the weather conditions allow it, train outdoors on different surfaces: asphalt, grass and even in the mountains. However, there are sports modalities in which it is necessary to train within a facility, and the treadmill is the only training alternative available due to its characteristics and the possibility of maintaining a certain speed and slope.

When comparing overground vs. treadmill, running on a treadmill results in higher stride frequencies and shorter stride lengths [19], being this parameters closely related to running economy. According to Hunter & Smith [20] novice runners select lower stride frequencies than the optimal one, while experienced runners choose unconsciously higher frequencies, optimizing energy expenditure and improving running economy. Moreover, running on treadmill produces lower tibial peak acceleration and impact rates compared to the overground running [7,13].

Nowadays, new treadmill designs, such as the curved non-motorized treadmill (cNMT), have demonstrated to be a valid and reliable tool for rehabilitation, training and laboratory based research [21,22,23]. cNMT have been designed to evaluate the strength, maximum speeds, and power of the athlete, allowing a more specific running evaluation. cNMT have a curved non-motorized surface, which requires the runner to impact the surface and propel the band with each stride [23,24]. The main difference compared to motorized treadmills (MT) is that cNMT allows participants to self-select the speed and allows a more valid and ecological laboratory assessment of running performance [25,26]. While different studies have been analysing the cNMT on sprints [27], endurance running [26], cardiometabolic demands [28,29] and team-sport running [30]; other studies have focused on physiological and perceptual variables comparing cNMT with MT [21,25], and overground running [23].

However, only few studies have observed biomechanical changes during walking [29,31] or running on cNMT [32], observing shorter stride length compared to MT [33]. In terms of impact accelerations, just one research have analysed tibial impact acceleration during running on cNMT vs. MT [34], but no study of the impact transmission from the tibia to the head was carried out.

To our knowledge, no previous research has analysed the effect on head and tibial accelerations during running on cNMT. Therefore, the aim of the present study was to analyse impact accelerations, spatio-temporal parameters and perceptual changes during running on cNMT vs. MT at different speeds in recreational runners. It was hypothesized that: (a) running on cNMT would reduce impact acceleration parameters, increasing stride frequency and reducing stride length in comparison with motorized treadmill running; and (b) the effect of running speed would affect impact acceleration, increasing when running at higher velocities.

## 2. Materials and Methods

### 2.1. Participants

Twenty-seven recreational runners: 22 males and 5 females (age 25 ± 7 years, height 170.30 ± 8.09 cm, body mass 64.4 ± 10.3 kg, running training 37 ± 19 km/week) agreed to participate in the study and gave written informed consent. Inclusion criteria included to be physically active (to run a minimum of twice a week in the last year), a training volume of at least 20 km per week, no history of lower limb injuries within the last six months, no suffering of heart failure, neurological or musculoskeletal disorders affecting normal locomotion and to not be taking medication that interferes with stability during running. Exclusion criteria included injury, surgery or illness within the previous six months, and overweight or obesity (BMI > 24.9 kg/m^2^). Based on previous studies and a general linear model (GLM) of two-way Repeated Measures design, a total sample size of 22 participants was needed to detect significant differences associated with a minimum detectable effect size (moderate) f = 0.253 (α = 0.05, β = 0.05, power = 0.952) for acceleration impact.

The study procedures complied with the Declaration of Helsinki and were approved by the University ethics committee (UV-INV-ETICA-1245207).

### 2.2. Study Protocol

**Figure 1:**
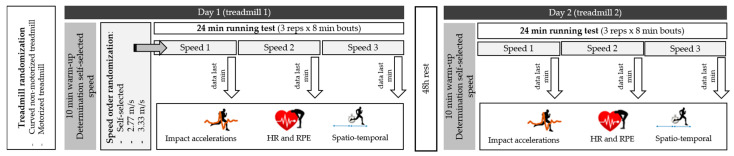
Schematic representation of the study protocol.

We carried out an experimental study with a quantitative approach without a control group and with a repeated measures design. Participants performed two randomised running test in different treadmills, one on cNMT (Bodytone ZRO-T, Bodytone International Sport S.L., Molina del Segura, Spain) and another on MT (h/p/cosmos pulsar^®^ 3p, h/p/cosmos sports & medical gmbh. Nußdorf, Germany) with 1% incline to replicate the energy cost of outdoor running [35]. Similarly on both surfaces, participants warmed-up for 10 min at self-selected speed, which also served as familiarization time on the treadmill [7]. Then, participants ran 24 min in three separated bouts of 8 min at 2.77 m/s, 3.33 m/s and self-selected speed, in a random order (Figure 1). A completely randomized design protocol, using opaque envelopes for allocation concealment, was used to the treadmill order selection, and running speed. Envelopes were equal in weight, similar in appearance, and tamperproof [36].

The self-selected speed was chosen by the participants during the warm-up for each treadmill and it was used then as a condition speed. Participants ran with their own shoes in both testing days to reduce biomechanics variability. The tests were separated by at least 48 h and were carried out at the same time of the day (±1 h).

Acceleration parameters were collected during the last minute of each bout with the purpose of reducing the measurement error due to stride variability [37]. A total of 2.430 strides on each treadmill and speed condition were analysed in the study. The rating of perceived exertion (RPE, 6–20 Borg scale) was reported after the end of each run [38]. Finally, heart rate (HR) was also registered during the last minute using a portable HR belt (Polar V800, Polar Electro, Kempele, Finland). Participants did not know neither the velocity that they were running nor the moment when measurements started or finished in order to avoid running alterations [39].

### 2.3. Data Collection

Acceleration parameters were measured by two lightweight triaxial wireless accelerometers (Pikkulab, Blautic Design, Valencia, Spain; total mass: 50 g; dimensions: 50 × 20 × 10 mm; range: ±16 g) firmly attached to the skin with double-sided adhesive tape [7]. Accelerometer-based analysis systems have been used routinely to continuously assess acceleration peaks during activities such as running and human gait, demonstrating excellent validity and reliability [10]. The accelerometers were placed on the forehead and the distal and anteromedial portion of the tibia [40] and secured by elastic belts. The vertical axis of the accelerometer was aligned to be parallel to the long axis of the shank, as the location of the tibial accelerometer does influence the acceleration signal [40]. Vertical acceleration data were registered at 180 Hz using software Pikkulab APP (Blautic Design, Valencia, Spain). For cNMT, distance running was registered during minutes 2–3, 4–5 and 6–7 to calculate the exact speed and spatio-temporal parameters were obtained from the impact accelerations and the exact speed.

Acceleration data were analysed using Matlab (MathWorks, MA, USA). For the spatio-temporal and impact acceleration analysis, the acceleration signal was filtered (Butterworth, second-order, low-pass, cut-off frequency = 50 Hz) and stride length, stride frequency, tibia and head acceleration rate (slope from ground contact to peak acceleration), tibia and head peak acceleration (maximum value of the acceleration signal), tibia and head acceleration magnitude (difference between the positive and the negative acceleration peak) and shock attenuation (reduction in impact acceleration from the tibia to the head) were calculated from the acceleration signal [40].

### 2.4. Statistical Analysis

Statistical analyses were carried out using SPSS.25 statistics software package (SPSS Inc., Chicago, IL, USA). The normality of the data was verified using the Shapiro–Wilk test (*p* = 0.274). Then, a general linear model of two-way repeated-measures design was performed. Post hoc comparisons were performed using the Bonferroni test to identify the location of specific differences. RPE and HR were analysed through a Friedman test. In those cases where significant differences were found (*p* < 0.05), the Wilcoxon test was performed for pairwise comparison. For parametric and non-parametric analysis, running treadmill (cNMT and MT) and running speed (2.77 m/s, 3.33 m/s and self-selected speed) were considered as within-subject factors. The level of significance was set at *p* < 0.05. For significant pair differences, Cohen’s effect sizes (ES) were computed and 95% confidence intervals of the differences (95% CI) were provided [41]. 

## 3. Results

No statistically significant differences (*p* > 0.05) were found between women and men. Therefore, data analysis was carried out as a homogeneous sample.

### 3.1. Treadmill Differences

**Figure 2:**
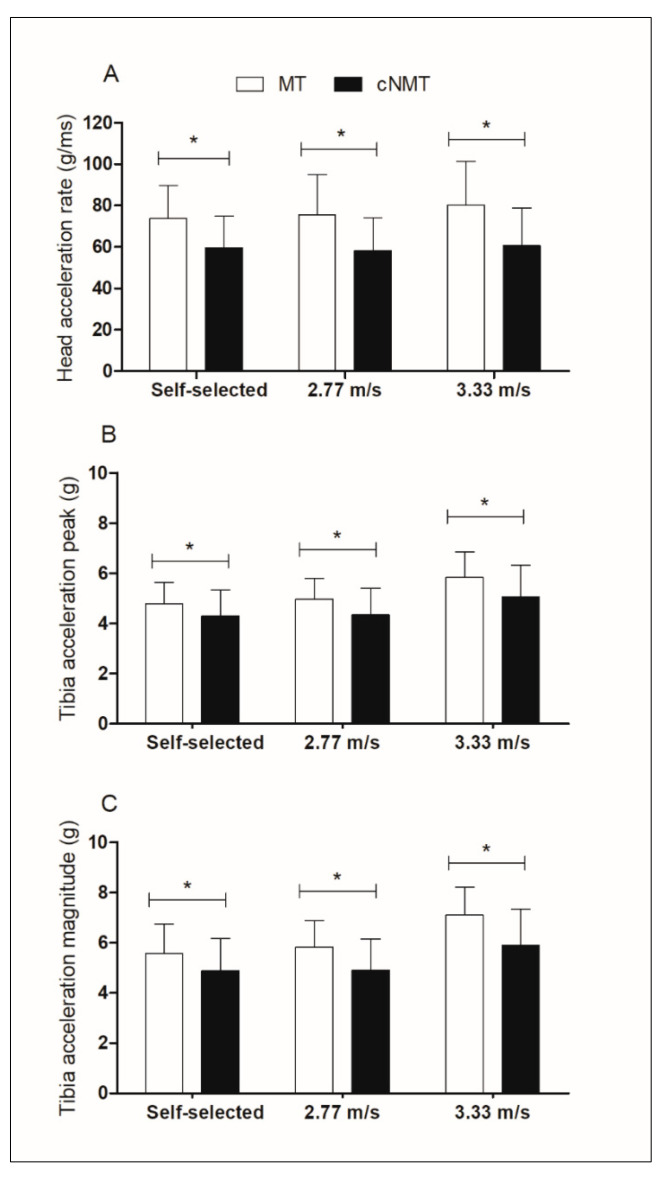
(**A**) Head acceleration rate (g/ms); (**B**) Tibial acceleration peak (g); (**C**) Tibia acceleration magnitude (g) at different speeds and surface. MT: motorized treadmill; cNMT: curved non-motorized treadmill. * Statistical differences (*p* < 0.05) between treadmills.

Running on cNMT provoked significantly lower impacts in head acceleration rate in comparison with MT at self-selected speed (*p* = 0.000, ES = 0.917, mean difference: 15.488, 95% CI [9.064–21.913]), 2.77 m/s (*p* = 0.000, ES = 0.706, mean difference: 20.422, 95% CI [11.378–29.467]) and 3.33 m/s (*p* = 0.000, ES = 0.951, mean difference: 23.463, 95% CI [13.311–33.615]) (Figure 2). In terms of tibia peak acceleration, differences between treadmills were observed at self-selected speed (*p* = 0.008, ES = 0.37, mean difference: 0.489, 95% CI [0.141–0.838]), 2.77 m/s (*p* = 0.001, ES = 0.477, mean difference: 0.685, 95% CI [0.338–1.033]) and 3.33 m/s (*p* = 0.001, ES = 0.495, mean difference: 0.865, 95% CI [0.420–1.309]) (Figure 2). Finally, differences in tibia acceleration magnitude at self-selected speed (*p* = 0.022, ES = 0.398, mean difference: 0.699, 95% CI [0.109–1.290]), 2.77 m/s (*p* = 0.001, ES = 0.568, 1.016, 95% CI [0.464–1.568]) and 3.33 m/s (*p* = 0.000, ES = 0.67, mean difference: 1.362, 95% CI [0.732–1.991]) were found when cNMT was compared to MT (Figure 2). However, no other statistically significant (*p* > 0.05) differences in impact accelerations were found between treadmill conditions (Table 1). 

Spatio-temporal parameters were not significantly different between treadmills in any of the conditions of the study (*p* > 0.05) (Table 2). Regarding the RPE, significantly higher values (*p* < 0.05) were found in cNMT compared to MT at self-selected speed (*p* = 0.035, ES = 0.356), 2.77 m/s (*p* = 0.032, ES = 0.338) and 3.33 m/s (*p* = 0.041, ES = 0.311) (Table 2). Likewise, HR was significantly higher on cNMT than on MT at self-selected speed (*p* = 0.011, ES = 0.413), 2.77 m/s (*p* = 0.000, ES = 0.62) and 3.33 m/s (*p* = 0.003, ES = 0.49).

### 3.2. Speed Differences

Comparing between running speeds on the same treadmill, impact accelerations were significantly higher (*p* < 0.05) when running at 3.33 m/s. Particularly, differences were observed in head rate acceleration between 2.77 m/s and 3.33 m/s (*p* = 0.026, ES = 0.232, mean difference: 3.649, 95% CI [6.934–0.364]). In terms of tibial peak acceleration, differences between self-selected speed and 3.33 m/s (*p* = 0.000, ES = 1.157, mean difference: 0.984, 95% CI [0.598–1.370]) and between 2.77 m/s and 3.33 m/s (*p* = 0.000, ES = 0.959, mean difference: 0.862, 95% CI [0.653–1.072]) were observed. Finally, we also found differences in tibial magnitude acceleration between self-selected speed and 3.33 m/s (*p* = 0.005, ES = 0.553, mean difference: 1.362, 95% CI [0.732–1.991]). However, no differences in other impact acceleration parameters could be observed (Table 1). 

Stride length increased as velocity was higher, finding significant (*p* < 0.05) differences between self-selected speed and 3.33 m/s (*p* = 0.000, ES = 1.723, mean difference: 371.244, 95% CI [253.304–489.184]), and between 2.77 m/s and 3.33 m/s (*p* = 0.000, ES = 2.777, mean difference: 334.927, 95% CI [239.046–430.808]). However, there were not significant differences (*p* > 0.05) in stride frequency when comparing running velocities (Table 2). In addition, significantly higher (*p* < 0.05) RPE and heart rate were observed when running speed increased, observing differences between self-selected speed and 3.33 m/s on MT (RPE: *p* = 0.000, ES = 0.68; HR: *p* = 0.000, ES = 0.591) and cNMT (RPE: *p* = 0.000, ES = 0.698; HR: *p* = 0.000, ES= 0.639) and between 2.77 m/s and 3.33 m/s on MT (RPE: *p* = 0.000, ES = 0.7; HR: *p* = 0.000, ES = 0.616) and cNMT (RPE: *p* = 0.000, ES = 0.684; HR: *p* = 0.000, ES = 0.622).

## 4. Discussion

The main objective of the present study was to analyse the influence of the treadmill system and speed on spatio-temporal, impact accelerations and perceptual parameters while running. To date, no studies have analysed head and tibia accelerations during running at different speeds on curved non-motorized treadmill in comparison with motorized treadmill. Based on the results achieved, we partially reject the null hypothesis since running on cNMT reduces impact acceleration parameters compared with MT, specifically in the parameters of head rate, tibial peak and tibial magnitude. While we cannot reject it in the parameters of stride frequency and stride length, as an increase in frequency and a reduction in stride length were not observed. Likewise, we reject the null hypothesis regarding the effect of speed on acceleration impacts, since they were significantly higher at higher speeds (3.33 m/s) than at lower speeds (2.77 and self-select speed).

Results of this study shows statistically significant (*p* < 0.05) reductions in head rate acceleration, tibia peak acceleration and tibia acceleration magnitude when participants ran on cNMT in comparison with MT, but no differences were found in other impact acceleration parameters. These reductions could be caused by the concave belt, which has a pronounced forward lean and favours forefoot striking instead of midfoot/heel striking [34].

Impact on tibial acceleration is related to lower limb fatigue injuries in runners and the risk for tibial stress fracture [13,15,40], and it has been studied at different running surfaces [12,13,14,15,41,42]. In light of this, Montgomery et al. [34] suggested that walking, jogging and running on the cNMT produces large reductions in tibial accelerations in comparison with overground and MT running. Nevertheless, other studies have not find any differences in tibial impact between a wide range of surfaces, as synthetic track, concrete, natural grass, MT or EVA treadmill [43]. 

Shock attenuation in the present study did not differ significantly between running surfaces (MT or cNMT). Conversely, Dufek et al. [16] showed significant differences in shock attenuation when it was compared between gender, speed (preferred and 10% slower) and surface (soft, medium and hard). It has been suggested that a reduction in shock attenuation caused by the running surface, fatigue or injuries can be harmful for the musculoskeletal system and increase the risk of injury [44]. 

According toBruseghini et al. [21], stride length and stride frequency were expected to differ between treadmills due to the belt friction, curvature and dimensions, but no significant changes (*p* > 0.05) were found in spatio-temporal parameters when both treadmills were compared. In accordance with this outcomes, Seneli et al. [45] neither found any differences in step length while walking, jogging or running on cNMT and MT. Different studies have analysed the influence of stride length and stride frequency on impact accelerations [46], where peak impact acceleration showed a negative linear trend as stride length increased [6,44]. Other investigations showed differences between treadmills in stride length and stride frequency while walking at preferred speed [21], and shorter stride length when participants ran on cNMT [32,34]. Moreover, it has been shown that stride frequency decreases after a 30 min fatiguing run [44]. 

Rating of perceived effort and heart rate were significantly (*p* < 0.05) higher while running on cNMT in comparison with MT. This fact could be because this type of treadmills require energy not only to drive the body itself, but also to drive the belt in every single step, to which friction and slope are attributed [21,25]. Different studies ensure that running on cNMT can produce greater perceived fatigue [22,28,29], allowing participants to obtain greater physiological benefits associated with moderate and vigorous exercise without any substantial increase in effort compared to MT [29]. 

Observing the differences between speeds, significantly (*p* < 0.05) higher head impact acceleration, tibial peak acceleration and tibial acceleration magnitude when running at 3.33 m/s, increasing while running speed increased either on MT or cNMT. These results are in line with those observed by Sheerin et al. [42], who found an increase 38% (3.8 g) in tibial acceleration from the slowest to the fastest velocities. However, only few studies have studied impact accelerations in the head and tibia during running at different speeds [47].

Head accelerations remained constants for every treadmill and speed condition, being lower than tibia acceleration. It has been suggested as a protective behavior to prevent a possible disruption of the visual and vestibular system that could occur due to the excessive head acceleration [7,11,45]. Moreover, differences in stride length have been observed between speed conditions, increasing linearly with speed, in agreement with previous studies [47].

RPE and HR were higher when running at 3.33 m/s, condition which was found to be more exhausting by the participants and produced higher intensity, being one point lower in Borg’s scale on MT. Furthermore, running on cNMT not only increase physiological demands due to the increment in intensity but also required regular adjustments to keep uniform velocity, either through speed and/or stride length; thus, cNMT probably also requires greater neuromuscular control than MT [29]. 

In summary, the results observed in the present study allow practical implications, showing that acceleration data were different when running on cNMT vs. MT. In addition, impact acceleration was influenced by treadmill and speed. Regarding the effort perception, the present study has shown important changes between cNMT and MT, with higher rating of perceived effort and heart rate while running in cNMT compared to MT.

## 5. Conclusions

In conclusion, running on cNMT reduces impact accelerations and produces higher heart rate and rating of perceived effort in comparison with MT, but no differences in spatio-temporal parameters were found between treadmills.

On the other hand, in relation to the speed effect on biomechanical variables, a logical increase was observed in impact accelerations, stride length, RPE and HR when running at 3.33 m/s compared to self-selected speed and 2.77 m/s. 

Therefore, as a practical application, running on a cNMT could become an interesting training tool for athletes, trainers, physiotherapist and researchers due to the loading reduction and the increased physiological and perceptual response. Curved non-motorized treadmill could be a good strategy inside return-to-play rehabilitation protocols, high intensity training sessions or simply for loading reduction on long distance training athletes.

## 6. Limitations

The present study is not without limitations. In the present study, only the dominant leg was analysed; the analysis of the two legs could provide information on the symmetry of the running cycle in both extremities.

Another possible limitation would be related to the characteristics of the sample and the adaptation time to the treadmill, since there are few runners with previous experience using a curved non-motorized treadmill. In our study, we have tried to minimize this bias by using a protocol in which the participants had enough time to adapt to the new condition.

Therefore, with the results obtained in our study, we believe that future lines of research should aim to analyse running on both types of treadmill (cNMT vs. MT) in both lower extremities, as well as analysing with larger samples from groups with differing levels of sports experience. Finally, it would be interesting to evaluate the effect of cNMT training on running technique.

## Figures and Tables

**Table 1:** Parameters (mean and standard deviation) based on speed and surface.

Parameters	Treadmill	Self-Selected (m/s)	2.77 m/s	3.33 m/s
**Head rate (g/ms)**	**MT**	73.62 (15.94) ^†^	75.49 (19.36) ^†,c^	80.12 (21.2) ^†,b^
	**cNMT**	59.57 (15.27)	58.09 (15.98) ^c^	60.61 (18.07) ^b^
**Tibial rate (g/ms)**	**MT**	247.26 (91.91)	257.74 (114.12)	313.19 (119.58)
	**cNMT**	257.63 (136.30)	268.58 (137.32)	307.10 (143.16)
**Head peak (g)**	**MT**	2.64 (0.3)	2.65 (0.28)	2.74 (0.29)
	**cNMT**	2.51 (0.31)	2.47 (0.25)	2.58 (0.30)
**Tibial peak (g)**	**MT**	4.78 (0.85) ^†,c^	4.97 (0.83) ^†,c^	5.84 (1.01) ^†,a,b^
	**cNMT**	4.29 (1.05) ^c^	4.34 (1.06) ^c^	5.06 (1.25) ^a,b^
**Head magnitude (g)**	**MT**	2.88 (0.36)	2.90 (0.34)	3.07 (0.34)
	**cNMT**	2.68 (0.37)	2.63 (0.29)	2.82 (0.36)
**Tibial magnitude (g)**	**MT**	5.56 (1.18) ^†,c^	5.81 (1.06) ^†^	7.10 (1.12) ^†,a^
	**cNMT**	4.88 (1.28) ^c^	4.90 (1.24)	5.90 (1.44) ^a^
**Attenuation (%)**	**MT**	42.73 (9.55)	45.14 (9.57)	51.93 (8.16)
	**cNMT**	38.89 (15.02)	40.28 (14.45)	46.71 (13.04)

MT: motorized treadmill; cNMT: curved non-motorized treadmill. ^†^ differences between treadmills (*p* < 0.05); ^a^ difference with self-selected speed (*p* < 0.05); ^b^ difference with 2.77 m/s (*p* < 0.05); ^c^ difference with 3.33 m/s (*p* < 0.05).

**Table 2:** Parameters (mean and standard deviation) based on speed and surface.

Parameters	Treadmill	Self-Selected (m/s)	2.77 m/s	3.33 m/s
**Stride length (m)**	**MT**	1.86 (0.25)	1.89 (0.10)	2.19 (0.12)
	**cNMT**	1.87 (0.21)	1.88 (0.13)	2.24 (0.28)
**Stride frequency (Hz)**	**MT**	1.47 (0.07)	1.47 (0.08)	1.52 (0.09)
	**cNMT**	1.51 (0.09)	1.51 (0.09)	1.51 (0.13)
**RPE**	**MT**	9.63 (1.90) ^†,c^	9.81 (2.27) ^†,c^	12.44 (2.44) ^†,a,b^
	**cNMT**	10.46 (1.65) ^c^	10.54 (2.08) ^c^	13.42 (2.79) ^a,b^
**Heart rate (bpm)**	**MT**	149.74 (17.35) ^†,c^	150.69 (17.11) ^†,c^	161.45 (16.77) ^†,a,b^
	**cNMT**	156.82 (17.01) ^c^	158.87 (15.81) ^c^	167.55 (17.93) ^a,b^

MT: motorized treadmill; cNMT: curved non-motorized treadmill; RPE: rating of perceived exertion. ^†^ differences between treadmills (*p* < 0.05); ^a^ differences with self-selected speed (*p* < 0.05); ^b^ difference with 2.77 m/s (*p* < 0.05); ^c^ difference with 3.33 m/s (*p* < 0.05).

## Data Availability

Not applicable.
